# Magnesium in Ketamine Administration in Treatment-Resistant Depression

**DOI:** 10.3390/ph14050430

**Published:** 2021-05-03

**Authors:** Natalia Górska, Wiesław Jerzy Cubała, Jakub Słupski, Mariusz Stanisław Wiglusz, Maria Gałuszko-Węgielnik, Mateusz Kawka, Agata Grzegorzewska

**Affiliations:** Department of Psychiatry, Faculty of Medicine, Medical University of Gdańsk, 80-952 Gdańsk, Poland; nataliagorska@gumed.edu.pl (N.G.); cubala@gumed.edu.pl (W.J.C.); jslupski@gumed.edu.pl (J.S.); mwiglusz@gumed.edu.pl (M.S.W.); mgaluszko@gumed.edu.pl (M.G.-W.); agata.grzegorz@wp.pl (A.G.)

**Keywords:** magnesium, ketamine, treatment-resistant depression

## Abstract

Relationship between depression and magnesium levels is reported. This observational study examined whether serum magnesium concentration change over time of ketamine treatment course, also whether association between magnesium concentrations and treatment response measured with Montgomery-Åsberg Depression Rating Scale (MADRS) score occurs. Moreover, interlink between changes in Young Mania Rating Scale (YMRS) score, somatic comorbidities, and magnesium concentration was studied. Inpatients with major depressive disorder or bipolar disorder were rated weekly by clinician using MADRS and YMRS. Magnesium levels assessments were carried out weekly, before start of ketamine treatment and then every second infusion and one week after last ketamine infusion. The concentration of Mg^2+^ ions differs depending on the measurement. The Mg^2+^ concentration in pre-measurement was significantly higher than in measurement after five infusions (*p* = 0.031) and after seven infusions (*p* = 0.003). No significant correlation was observed between changes in magnesium serum levels and MADRS or YMRS. The concentration of Mg^2+^ ion in course of the treatment was not associated with somatic comorbidities. The study supports data for role of magnesium in treatment-resistant depression, particularly related to ketamine treatment, but provides no clear evidence of straightforward association between magnesium serum concentration and treatment response or comorbidity.

## 1. Introduction

Depression is one of the major causes of disability, affecting more than 300 million people worldwide, among whom 10–30% of patients fail to achieve remission with index pharmacological treatment [1,2]. Treatment-resistant depression (TRD) is common in major depressive disorder (MDD) and bipolar disorder (BP), being a challenging clinical condition and serious overall issue [3]. Current antidepressant treatment (ADT) includes drugs exhibiting a homogeneous mechanism of action mainly related to the regulation of the monoaminergic systems, with unmet needs in non-monoaminergic mechanisms of action [2,4]. Pathophysiology of depression is complex and it also incorporates glutamatergic system and magnesium ions imbalance [5,6,7].

Ketamine is a promising drug, as there is evidence of its tolerability, safety, and efficacy in TRD [8]. Ketamine exhibits a rapid antidepressive effect through the glutamatergic system by its antagonistic effect of N-methyl-D-aspartate receptor (NMDAR) with magnesium (Mg^2+^) as a co-factor [9,10,11]. Multiple studies have observed a relationship between depression and magnesium levels [12,13]. Studies indicate the antidepressive properties of magnesium and its potential usage as an additive substance to antidepressant treatment [14,15]. Magnesium ions contribute to neurotransmission, in particular glutamatergic transmission, and several reports indicate the possible role of magnesium imbalance in the pathophysiology of mood disorders [16,17]. Although no direct relationship was found, magnesium levels seem to be associate with a chronic and treatment-resistant course of depression. It has been hypothesized that the synergistic effect between the pharmacodynamics of magnesium and ketamine in the treatment of depression impacts the antidepressive effect of ketamine infusions in patients with mood disorders [9,18].

This paper explores the associations of blood Mg^2+^ levels and the effect of intravenous ketamine infusions for the treatment of MDD and BD patients in an open-label naturalistic study.

## 2. Results

**Figure 1:**
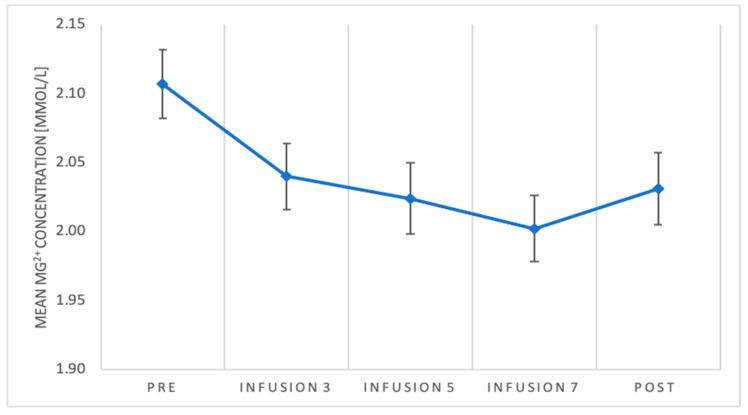
Mean Mg^2+^ concentration and standard errors of the mean for the concentration of Mg^2+^ in individual measurements.

**Figure 2:**
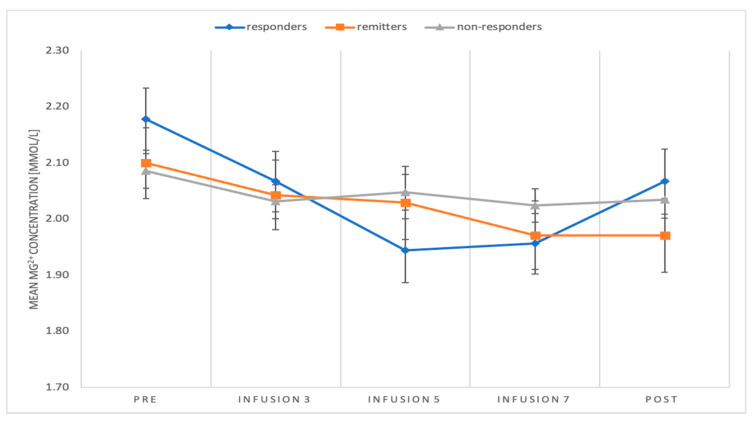
Mean Mg^2+^ concentration and standard errors of the mean for the concentration of Mg^2+^ ions in individual measurements.

Demographics and clinical characteristics are presented in Table 1. The concentration of Mg^2+^ ions differs depending on the measurement, *F*(4) = 5.22; *p* = 0.001; *η2p* = 0.11. The Mg^2+^ concentration in pre-measurement was significantly higher than in measurement after fifth infusion (*p* = 0.031) and after seventh infusion (*p* = 0.003), but it was non-significant for third infusion measurement (*p* = 0.126) and post-measurement (*p* = 0.051) (Figure 1). Analysis of simple effects for Mg concentration showed significant differences only in responders group, *F* (4.39) = 6.26; *p* = 0.001; *η2p* = 0.39. In the responders’ group, the Mg level in the pre-measurement was significantly higher than in the measurement after the fifth infusion (*p* = 0.001) and after the seventh infusion (*p* = 0.003). It did not differ significantly from the measurement after the third infusion (*p* = 0.628) and in the post-measurement (*p* = 0.606) (Figure 2). We did not find any correlation between MADRS score and Mg^2+^ concentration. For TRD in MDD and BP subjects, magnesium concentration did not significantly change during the treatment. The concentration of Mg ions in the course of the treatment was also not associated neither with number of somatic comorbidities, nor with specific comorbidities, such as: arterial hypertension, diabetes mellitus, hyperlipidemia, post-stroke condition, and epilepsy (Table 2). We also did not find statistical significance between gender differences in responder/remitter/non-responder group (chi-square test = 0.1877, *p* = 0.6648).

## 3. Discussion

In the present study, we did not find any correlation between magnesium concentrations and the treatment outcome measures in the course of intravenous ketamine administration in patients with TRD. Also, somatic comorbidities did not impact magnesium levels in the study group.

In preclinical studies, magnesium administered together with a low dose NMDA antagonist caused a reduction in depressive-like behaviors during the animal depression model test-FST (forced swim test) in mice [17,19]. One animal study hypothesized about magnesium potentially being a co-factor that would increase the effect of ketamine but not confirm the usefulness of magnesium as a supportive treatment for ketamine in depression in mice [7]. Other studies suggested magnesium supplementation to antidepressants—such as imipramine, fluoxetine, citalopram, tianeptine, and bupropion—resulted in a synergistic antidepressant effect in FST paradigm [20,21]. Moreover, in rats, the sensitivity to ketamine and other NMDAR antagonists increases in the state of magnesium depletion [22]. Some observations indicate that the combination of ketamine and magnesium in an average magnesium level has a superadditive effect [23].

The concentration of magnesium in serum may be a potential marker in patients with depression. Most studies exhibit low magnesium levels in depression [16,24]; however, a limited number of studies suggest the opposite [12,25]. Multiple clinical studies indicate the antidepressant properties of magnesium and its potential usage as adjunctive therapy to antidepressant treatment [14,23,26,27,28]. Interestingly one study demonstrated an increase in intracellular magnesium concentration after the treatment with antidepressants (amitriptyline, sertraline) [29]. According to several recent studies, ketamine demonstrated a robust antidepressant effect acting via the glutamatergic pathway. Ketamine exhibits a rapid antidepressant by its antagonistic effect on NMDAR with magnesium as a co-factor [10,11]. Ketamine also acts as a weak agonist at *mu* opioid receptors [30] and activates dopamine release [31]. The neurochemical features of ketamine depend on the isoform present in an interaction with the receptor. (S)—ketamine has greater affinity for the NMDAR, whilst (R)—ketamine has greater opioid action [32]. The mechanism underlying the efficacy of ketamine in depression is thought to be related to increased neuroplasticity [33]. Fluctuations in the metabolic activity of the hippocampus, dorsal anterior cingulate cortex, and orbital-frontal cortex in combination with altered reward processing activity have been observed in patients treated with ketamine [34]. It has also been shown that ketamine increases dopamine levels in the central nervous system, which results in a reduction in D2-receptor expression as a potential compensatory mechanism for excessive dopamine stimulation [35]. Ketamine appears to be safe and well-tolerated drug as the majority of adverse drug reactions are mild and tend to disappear within 30 min to 2 h after ketamine administration, including cardiovascular and dissociative symptoms [36].

The synergistic effect between the pharmacodynamic activity of magnesium and ketamine in the treatment of depression may play an important role in depression treatment [9,18]. Ketamine also directly or indirectly increases magnesium levels in the brain by activating non-NMDA glutamatergic receptors [9]. Magnesium is involved in many mechanisms that are key to the pathophysiology of major depression, which is also affected by ketamine administration. However, the results concerning the influence of magnesium on ketamine efficacy are inconclusive.

## 4. Materials and Methods

### 4.1. Subjects

The study population comprises subjects enrolled in a naturalistic safety and efficacy registry protocol for ketamine infusions in TRD. We enrolled hospitalized patients diagnosed with a depressive episode in the course of MDD and BP. According to the Diagnostic and Statistical Manual of Mental Disorders (DSM-5) criteria, a clinician psychiatrist interviewed patients to establish the diagnosis using a Mini-International Neuropsychiatric Interview (MINI). All participants met the criteria for TRD, defined as an inadequate response to two or more antidepressants (assessed by Massachusetts General Hospital Antidepressant Treatment Response Questionnaire–ATRQ) in the course of treatment of that particular episode. The study followed the rule of single-patient and single-rater. In the screening period, patients were rated by the same clinician using MADRS and YMRS. Only medically stable, able to communicate and provide written informed consent, adult inpatients aged 18–65 were enrolled to study. We performed standard of care procedures (physical examination, medical history, vital signs, laboratory tests, electrocardiography). Patients continued current medication during ketamine treatment. The exclusion criteria included a history of uncontrolled medical conditions, a previous adverse reaction to ketamine, active substance use, pregnancy, or breastfeeding.

The study was carried out according to the Declaration of Helsinki, Fortaleza amendment. The study protocol was approved by the institutional review board (IRB). We obtained written informed consent for each participant after explaining all procedures related to the study.

### 4.2. Study Protocol

The study followed an observational design. The patients continued baseline psychotropic drugs and necessary treatment of chronic somatic diseases during ketamine infusions. The study’s therapeutic intervention was based on the administration of eight ketamine infusions over four weeks. Ketamine was dosed at 0.5 mg/kg based on the patient’s actual body weight and infused intravenously over 40 min. Safety monitoring was performed by the same attending psychiatrist before, during, and post-infusion every 15 min up to an hour and a half post-infusion, including a periodic assessment of vital signs (heart rate, body temperature, respiratory rate, blood pressure, oxygen saturation). The ECG, magnesium level, psychometric assessments with MADRS and YMRS, was assessed before the first, third, fifth, and seventh infusion and one week after the last infusion.

Responders were defined as an at least 50% improvement from baseline in MADRS total score and remitters if the MADRS total score was ≤10 points [37].

### 4.3. Assays

The magnesium level was determined by Abbott Alinity c enzymatic assay for magnesium in serum. The limit of detection was 0.07 mmol/dL. The measurement values in serum remained linear in the whole measurement spectrum ranging from 0.25 to 3.91 mmol/dL, based on allowed tolerance criteria of 0.04 mmol/dL. Blood samples were collected into tubes containing heparin and centrifuged at 4000 rpm with serum send for assay.

### 4.4. Statistical Analysis

We used Shapiro–Wilk test to assess the normal distribution of continuous data. Continuous data were compared with nonparametric tests lacking normal distribution with an alpha = 0.05. The comparison of measurements executed was performed using the Wilcoxon Rank Mark Test for Mg^2+^ levels, diagnosis, MADRS, YMRS, and comorbidity. We performed an analysis of variance with repeated measurements to determine the dynamics of changes in Mg^2+^ and psychometric variables during the infusion. The Mauchly test was employed to confirm the null hypothesis on the proportionality of the error covariance matrix. We also performed a post hoc analysis using the Bonferroni test to determine which measurements showed a statistically significant difference.

## 5. Limitations and Conclusions

The present study should be interpreted in light of the following limitations. The study may be underpowered regarding the small sample size. The research was performed as a single-site study. Each patient was rated by the same physician throughout the study. Raters were trained and tested by qualified trainee in applied scales. The differences in rating were discussed and corrected during training. However, no interrater reliability was measured. There was no treatment blinding during this observational protocol study. The observations apply to treatment-resistant patients and include unipolar and bipolar depressed patients with or without somatic disorders and psychiatric comorbidities and represent real-world clinical practice. We analyzed the magnesium levels extracted from peripheral blood serum based on the positive correlation between the central and peripheral Mg^2+^ levels [38]. Magnesium was extracted only from serum, not any other bodily fluids. Mg^2+^ was measured with an enzymatic test, not with the typically preferred absorption spectrometry test. We chose the enzymatic systems to overcome the interference by heavy metals, proteins, and lipemia related factors (problematic for chemically-based colorimetric systems) [39], which could not be controlled in the naturalistic protocol design. One measure per sample was performed. Neither intra-, nor inter-assay coefficients were measured.

The study does not support the evidence for ketamine intravenous add-on treatment and normalization of magnesium ions in TRD responders, signaling the possible interlink between ketamine administration and magnesium concentration in the active phase of treatment.

## Figures and Tables

**Table 1:** Demographics and clinical profile.

			*N*	Responder	Remitter	Non-Responder	*p*	*V*
Male	(%)		21 (42.9)	6 (66.7)	2 (25.0)	13 (40.6)	0.229	0.26
Female	(%)		28 (57.1)	3 (33.3)	6 (75.0)	19 (59.4)		
Mean age, in years			50.02	53.11	42.88	50.94	0.336	0.00
Occupation							0.250	0.32
	Employed		11 (22.4)	2 (22.2)	2 (25.0)	7 (21.9)		
	Unemployed		27 (55.1)	7 (77.8)	3 (37.5)	18 (56.2)		
	Pension		10 (20.4)	0 (0)	3 (37.5)	7 (21.9)		
	Seasonal work		1 (2.0)	0 (0)	0 (0)	1 (3.1)		
Education							0.252	0.26
	Elementary or lower		2 (4.1)	0 (0)	1 (12.5)	1 (3.1)		
	Vocational		5 (10.2)	2 (22.2)	1 (12.5)	2 (6.3)		
	Secondary		18 (36.7)	1 (11.1)	3 (37.5)	14 (43.8)		
	Higher		24 (49.0)	6 (66.7)	3 (37.5)	15 (46.9)		
BMI			27.92 (5.67)	28.00 (4.64)	26.50 (4.72)	28.25 (6.21)	0.613	0.02
Ketamine treatment for:								
	MDD		35 (71.4)	8 (88.9)	5 (62.5)	22 (68.8)	0.475	0.19
	BP		14 (28.6)	2 (11.1)	5 (37.5)	7 (31.2)	0.485	0.18
Comorbidity							0.104	0.31
		1	21 (42.9)	6 (66.7)	2 (25.0)	13 (40.6)		
		2	10 (20.4)	2 (22.2)	1 (12.5)	7 (21.9)		
		3	4 (8.2)	1 (11.1)	2 (25.0)	1 (3.1)		
	Arterial hypertension		16 (32.7)	6 (66.7)	3 (37.5)	7 (21.9)	0.037	0.37
		BP	4 (8.2)	1 (11.1)	2 (25.0)	1 (3.1)	0.052	0.66
		MDD	12 (24.5)	5 (55.6)	1 (12.5)	6 (18.8)	0.177	0.33
	Diabetes mellitus		3 (6.1)	1 (11.1)	2 (25.0)	0 (0)	0.021	0.39
	Hyperlipidaemia		9 (18.4)	3 (33.3)	1 (12.5)	5 (15.6)	0.545	0.19
	Post-stroke		3 (6.1)	1 (11.1)	0 (0)	2 (6.3)	0.731	0.14
	Post-myocardial infarction		0 (0)	0 (0)	0 (0)	0 (0)	-	-
	Epilepsy		6 (12.2)	0 (0)	3 (37.5)	3 (9.4)	0.060	0.36
	Other		16 (32.7)	2 (22.2)	1 (12.5)	13 (40.6)	0.330	0.24
Coexisting treatment								
	TCA		8 (16.3)	1 (11.1)	1 (13.5)	6 (18.8)	1.000	0.09
	Clomipramine		4 (8.2)	0 (0)	1 (12.5)	3 (9.4)	0.789	0.15
	Amitriptyline		4 (8.2)	1 (11.1)	0 (0)	3 (9.4)	1.000	0.13
	SSRI total		23 (46.9)	5 (55.6)	2 (25.0)	16 (50.0)	0.413	0.20
	Fluvoxamine		1 (2.0)	0 (0)	0 (0)	1 (3.1)	1.000	0.11
	Paroxetine		5(10.2)	1 (11.1)	0 (0)	4 (12.5)	0.813	0.15
	Fluoxetine		8 (16.3)	2 (22.2)	0 (0)	6 (18.8)	0.534	0.20
	Sertraline		3 (6.1)	1 (11.1)	0 (0)	2 (6.3)	0.731	0.14
	Citalopram		4 (8.2)	0 (0)	2 (25.0)	2 (6.3)	0.179	0.29
	Escitalopram		2 (4.1)	1 (11.1)	0 (0)	1 (3.1)	0.578	0.18
	SNRI total		11 (22.4)	2 (22.2)	2 (25.0)	7 (21.9)	1.000	0.03
	Venlafaxine		8 (16.3)	1 (11.1)	1 (12.5)	6 (18.8)	1.000	0.10
	Duloxetine		3 (6.1)	1 (11.1)	1 (12.5)	1 (3.1)	0.273	0.17
	Other ADTs:						0.749	0.14
		1	15 (30.6)	4 (44.4)	2 (25.0)	9 (28.1)		
		2	3 (6.1)	0 (0)	1 (12.5)	2 (6.3)		
	Mirtazapine		9 (18.4)	2 (22.2)	1 (12.5)	6 (18.8)	1.000	0.08
	Mianserin		3 (6.1)	1 (11.1)	0 (0)	2 (6.3)	0.731	0.14
	Trazodone		4 (8.2)	1 (11.1)	1 (12.5)	2 (6.3)	0.432	0.10
	Bupropion		3 (6.1)	0 (0)	1 (12.5)	2 (6.3)	0.488	0.15
	Vortioxetine		2 (4.1)	0 (0)	1 (12.5)	1 (3.1)	0.333	0.20
	Antipsychotics						0.806	0.15
		1	12 (24.5)	2 (22.2)	1 (12.5)	9 (28.1)		
		2	5 (10.2)	0 (0)	1 (12.5)	4 (12.5)		
	Aripiprazole		6 (12.2)	0 (0)	1 (12.5)	5 (15.6)	0.685	0.18
	Quetiapine		10 (20.4)	1 (11.1)	1 (12.5)	8 (25.0)	0.668	0.16
	Olanzapine		5 (10.2)	1 (11.1)	1 (12.5)	3 (9.4)	1.000	0.04
	Risperidone		1 (2.0)	0 (0)	0 (0)	1 (3.1)	1.000	0.11
	Mood stabilisers						0.348	0.29
		1	15 (30.6)	2 (22.2)	4 (50.0)	9 (28.1)		
		2	6 (12.2)	1 (11.1)	0 (0)	5 (15.6)		
		3	1 (2.0)	0 (0)	1 (12.5)	0 (0)		
	Lithium		5 (10.2)	0 (0)	1 (12,5)	4 (12.5)	0.643	0.16
	Valproate		9 (18.4)	2 (22.2)	3 (37.5)	4 (12.5)	0.160	0.24
	Lamotrigine		7 (14.3)	1 (11.1)	1 (12.5)	5 (15.6)	1.000	0.05

**Table 2:** Mg^2+^ serum concentration vs. comorbidity.

Comorbidity	*F*	*df*	*p*	*η* ^2^ *_p_*
No. of comorbidities	1.38	12	0.182	0.09
Arterial hypertension	0.45	4	0.771	0.01
Diabetes mellitus	0.60	4	0.664	0.01
Hyperlipidemia	0.48	4	0.750	0.01
Stroke	1.89	4	0.114	0.04
Epilepsy	1.45	4	0.220	0.03
Other	0.33	4	0.855	0.01

## Data Availability

Data available upon request for non-commercial academic research.

## References

[1] Gelenberg A.J. (2010). The prevalence and impact of depression. J. Clin. Psychiatry.

[2] McIntyre R.S., Filteau M.J., Martin L., Patry S., Carvalho A., Cha D.S., Barakat M., Miguelez M. (2014). Treatment-resistant depression: Definitions, review of the evidence, and algorithmic approach. J. Affect. Disord..

[3] Ionescu D.F., Rosenbaum J.F., Alpert J.E. (2015). Pharmacological approaches to the challenge of treatment-resistant depression. Dialogues Clin. Neurosci..

[4] Gaynes B., Warden D., Trivedi M., Wisniewski S., Fava M., Rush A.J. (2009). What Did STAR*D Teach Us? Results From a Large-Scale, Practical, Clinical Trial for Patients With Depression. Psychiatr. Serv..

[5] Berman R.M., Cappiello A., Anand A., Oren D.A., Heninger G.R., Charney D.S., Krystal J.H. (2000). Antidepressant effects of ketamine in depressed patients. Biol. Psychiatry.

[6] Zarate C.A., Singh J.B., Carlson P.J., Brutsche N.E., Ameli R., Luckenbaugh D.A., Charney D.S., Manji H.K. (2006). A randomized trial of an N-methyl-D-aspartate antagonist in treatment-resistant major depression. Arch. Gen. Psychiatry.

[7] Razmjou S., Litteljohn D., Rudyk C., Syed S., Clarke M., Pentz R., Dwyer Z., Hayley S. (2016). The interactive effects of ketamine and magnesium upon depressive-like pathology. Neuropsychiatr. Dis. Treat..

[8] Serafini G., Howland R., Rovedi F., Girardi P., Amore M. (2014). The Role of Ketamine in Treatment-Resistant Depression: A Systematic Review. Curr. Neuropharmacol..

[9] Murck H. (2013). Ketamine, magnesium and major depression—From pharmacology to pathophysiology and back. J. Psychiatr. Res..

[10] Coyle C.M., Laws K.R. (2015). The use of ketamine as an antidepressant: A systematic review and meta-analysis. Hum. Psychopharmacol..

[11] Kishimoto T., Chawla J.M., Hagi K., Zarate C.A., Kane J.M., Bauer M., Correll C.U. (2016). Single-dose infusion ketamine and non-ketamine N-methyl-d-aspartate receptor antagonists for unipolar and bipolar depression: A meta-analysis of efficacy, safety and time trajectories. Psychol. Med..

[12] Cubała W.J., Landowski J., Szyszko M., Czarnowski W. (2014). Magnesium in drug-naïve patients with a short-duration, first episode of major depressive disorder: Impact on psychopathological features. Magnes. Res..

[13] Serefko A., Szopa A., Poleszak E. (2016). Magnesium and depression. Magnes. Res..

[14] Tarleton E.K., Littenberg B., MacLean C.D., Kennedy A.G., Daley C. (2017). Role of magnesium supplementation in the treatment of depression: A randomized clinical trial. PLoS ONE.

[15] Tarleton E.K., Littenberg B. (2015). Magnesium intake and depression in adults. J. Am. Board Fam. Med..

[16] Cheungpasitporn W., Thongprayoon C., Mao M.A., Srivali N., Ungprasert P., Varothai N., Sanguankeo A., Kittanamongkolchai W., Erickson S.B. (2015). Hypomagnesaemia linked to depression: A systematic review and meta-analysis. Intern. Med. J..

[17] Poleszak E., Wlaź P., Kedzierska E., Nieoczym D., Wróbel A., Fidecka S., Pilc A., Nowak G. (2007). NMDA/glutamate mechanism of antidepressant-like action of magnesium in forced swim test in mice. Pharmacol. Biochem. Behav..

[18] Górska N., Cubała W.J., Słupski J., Gałuszko-Węgielnik M. (2018). Ketamine and magnesium common pathway of antidepressant action. Magnes. Res..

[19] Singewald N., Sinner C., Hetzenauer A., Sartori S.B., Murck H. (2004). Magnesium-deficient diet alters depression- and anxiety-related behavior in mice—Influence of desipramine and Hypericum perforatum extract. Neuropharmacology.

[20] Szewczyk B., Szopa A., Serefko A., Poleszak E., Nowak G. (2018). The role of magnesium and zinc in depression: Similarities and differences. Magnes. Res..

[21] Poleszak E., Wlaź P., Szewczyk B., Kȩdzierska E., Wyska E., Librowski T., Szymura-Oleksiak J., Fidecka S., Pilc A., Nowak G. (2005). Enhancement of antidepressant-like activity by joint administration of imipramine and magnesium in the forced swim test: Behavioral and pharmacokinetic studies in mice. Pharmacol. Biochem. Behav..

[22] Begon S., Pickering G., Eschalier A., Mazur A., Rayssiguier Y., Dubray C. (2001). Role of spinal NMDA receptors, protein kinase C and nitric oxide synthase in the hyperalgesia induced by magnesium deficiency in rats. Br. J. Pharmacol..

[23] Orser B., Smith D., Henderson S., Gelb A. (1997). Magnesium deficiency increases ketamine sensitivity in rats. Can. J. Anaesth..

[24] Islam M.R., Islam M.R., ShalahuddinQusar M.M.A., Islam M.S., Kabir M.H., Mustafizur Rahman G.K.M., Islam M.S., Hasnat A. (2018). Alterations of serum macro-minerals and trace elements are associated with major depressive disorder: A case-control study. BMC Psychiatry.

[25] Styczeń K., Siwek M., Sowa-Kućma M., Dudek D., Reczyński W., Szewczyk B., Misztak P., Topór-Mądry R., Opoka W., Nowak G. (2015). The serum magnesium concentration as a potential state marker in patients with unipolar affective disorder. Psychiatr. Pol..

[26] Cardoso C.C., Lobato K.R., Binfaré R.W., Ferreira P.K., Rosa A.O., Santos A.R.S., Rodrigues A.L.S. (2009). Evidence for the involvement of the monoaminergic system in the antidepressant-like effect of magnesium. Prog. Neuro-Psychopharmacol. Biol. Psychiatry.

[27] Barragán-Rodríguez L., Rodríguez-Morán M., Guerrero-Romero F. (2008). Efficacy and safety of oral magnesium supplementation in the treatment of depression in the elderly with type 2 diabetes: A randomized, equivalent trial. Magnes. Res..

[28] Eby G.A., Eby K.L. (2006). Rapid recovery from major depression using magnesium treatment. Med. Hypotheses.

[29] Nechifor M. (2009). Magnesium in major depression. Magnes. Res..

[30] Oye I., Paulsen O., Maurset A. (1992). Effects of ketamine on sensory perception: Evidence for a role of N- methyl-D-aspartate receptors. J. Pharmacol. Exp. Ther..

[31] Rabiner E.A. (2007). Imaging of striatal dopamine release elicited with NMDA antagonists: Is there anything there to be seen?. J. Psychopharmacol..

[32] Morgan C.J.A., Curran H.V. (2012). Ketamine use: A review. Addiction.

[33] Gałuszko-Węgielnik M., Wiglusz M.S., Słupski J., Szałach Ł., Włodarczyk A., Górska N., Szarmach J., Jakuszkowiak-Wojten K., Wilkowska A., Cubała W.J. (2019). Efficacy of ketamine in bipolar depression: Focus on anhedonia. Psychiatr. Danub..

[34] Lally N., Nugent A.C., Luckenbaugh D.A., Ameli R., Roiser J.P., Zarate C.A. (2014). Anti-anhedonic effect of ketamine and its neural correlates in treatment-resistant bipolar depression. Transl. Psychiatry..

[35] Cao B., Zhu J., Zuckerman H., Rosenblat J.D., Brietzke E., Pan Z., Subramanieapillai M., Park C., Lee Y., McIntyre R.S. (2019). Pharmacological interventions targeting anhedonia in patients with major depressive disorder: A systematic review. Prog. Neuro-Psychopharmacol. Biol. Psychiatry.

[36] Włodarczyk A., Cubała W.J. (2020). Safety and tolerability of ketamine use in treatment-resistant bipolar depression patients with regard to central nervous system symptomatology: Literature review and analysis. Medicina.

[37] Trivedi M.H., Corey-Lisle P.K., Guo Z., Lennox R.D., Pikalov A., Kim E. (2009). Remission, response without remission, and nonresponse in major depressive disorder: Impact on functioning. Int. Clin. Psychopharmacol..

[38] Romeo V., Cazzaniga A., Maier J.A.M. (2019). Magnesium and the blood-brain barrier in vitro: Effects on permeability and magnesium transport. Magnes. Res..

[39] Ryan M.F., Barbour H. (1998). Magnesium measurement in routine clinical practice. Ann. Clin. Biochem..

